# Chemical conversion of cisplatin and carboplatin with histidine in a model protein crystallized under sodium iodide conditions

**DOI:** 10.1107/S2053230X14013995

**Published:** 2014-08-29

**Authors:** Simon W. M. Tanley, John R. Helliwell

**Affiliations:** a School of Chemistry, Faculty of Engineering and Physical Sciences, University of Manchester, Brunswick Street, Manchester M13 9PL, England

**Keywords:** cisplatin, carboplatin, sodium iodide crystallization, histidine, transiodoplatin (PtI_2_*X*_2_), PtI_3_*X* species

## Abstract

Cisplatin and carboplatin are platinum anticancer agents that are used to treat a variety of cancers. Previous X-ray crystallographic studies of carboplatin binding to histidine in hen egg-white lysozyme (HEWL) showed a partial chemical conversion of carboplatin to cisplatin owing to the high sodium chloride concentration used in the crystallization conditions. Also, the co-crystallization of HEWL with carboplatin in sodium bromide conditions resulted in the partial conversion of carboplatin to the transbromoplatin form, with a portion of the cyclobutanedicarboxylate (CBDC) moiety still present. The results of the co-crystallization of HEWL with cisplatin or carboplatin in sodium iodide conditions are now reported in order to determine whether the cisplatin and carboplatin converted to the iodo form, and whether this took place in a similar way to the partial conversion of carboplatin to cisplatin in NaCl conditions or to transbromoplatin in NaBr conditions as seen previously. It is reported here that a partial chemical transformation has taken place to a transplatin form for both ligands. The NaI-grown crystals belonged to the monoclinic space group *P*2_1_ with two molecules in the asymmetric unit. The chemically transformed cisplatin and carboplatin bind to both His15 residues, *i.e.* in each asymmetric unit. The binding is only at the N^δ^ atom of His15. A third platinum species is also seen in both conditions bound in a crevice between symmetry-related molecules. Here, the platinum is bound to three I atoms identified based on their anomalous difference electron densities and their refined occupancies, with the fourth bound atom being a Cl atom (in the cisplatin case) or a portion of the CBDC moiety (in the carboplatin case).

## Abstract 2

Crystals of HEWL with cisplatin and HEWL with carboplatin grown in sodium iodide conditions both show a partial chemical transformation of cisplatin or carboplatin to a transiodoplatin (PtI_2_ *X*_2_) form. The binding is only at the N^δ^ atom of His15. A further Pt species (PtI_3_ *X*) is also seen, in both cases bound in a crevice between symmetry-related protein molecules.

## 1. Introduction   

Cisplatin and carboplatin are platinum anticancer drugs that have long been used as cytostatics (http://www.cancerresearchuk.org/cancer-help/about-cancer/cancer-questions/what-are-cytostatic-drugs) in the fight against cancer by targeting DNA. Our previous X-ray crystallographic studies of carboplatin with HEWL, a model protein, have shown partial chemical conversion to cisplatin owing to the high sodium chloride conditions used in the crystallization mixture, as confirmed by the presence of extra anomalous difference density in the binding site that is not attributable to the Pt atom (Tanley *et al.*, 2013 ▶). Subsequently, we replaced sodium chloride with sodium bromide in the crystallization mixture to more easily observe the conversion of carboplatin to the bromo form (Tanley, Diederichs *et al.*, 2014 ▶). In this condition, the observed chemical transformation which took place was to the transbromoplatin form, and contrasted with the sodium chloride case where cisplatin was seen (Tanley *et al.*, 2013 ▶). Since a portion of the cyclobutanedicarboxylate (CBDC) moiety was still visible in the His15 ligand-binding site under the sodium bromide conditions, this confirmed that the chemical conversion of carboplatin to transbromoplatin was partial (Tanley, Diederichs *et al.*, 2014 ▶).

In the study reported here, cisplatin or carboplatin was co-crystallized with HEWL under sodium iodide conditions. The motivation for this new study is twofold. Firstly, to again confirm whether a chemical conversion occurs, and specifically whether it is to the *trans*-platinated or *cis*-platinated form and whether it is a partial or a full chemical conversion. Secondly, if an iodinated platinated species is observed then it would suggest the prospect of undertaking a dual *K*-edge (platinum and iodine) targeted radiation therapy experiment against a tumour. Cisplatin administration has previously been studied in the case of radiation therapy using a synchrotron source and the Pt *K* edge alone (Biston *et al.*, 2004 ▶; see also http://www.esrf.eu/UsersAndScience/Publications/Highlights/2004/Imaging/Ima10/). Sodium iodide has been used previously to crystallize native HEWL and in these conditions HEWL crystallizes in space group *P*2_1_ with two protein molecules in the asymmetric unit (Alderton & Fevold, 1946 ▶; Steinrauf, 1959 ▶, 1998 ▶).

## 2. Methods   

### 2.1. Crystallization conditions   

Co-crystallization of HEWL with cisplatin in NaI solution was carried out under similar conditions as published in Alderton & Fevold (1946 ▶) and Steinrauf (1959 ▶, 1998 ▶) but with 20 mg HEWL co-crystallized with 1.2 mg cisplatin in 75 µl DMSO, 462.5 µl 0.1 *M* sodium acetate, 462.5 µl 1 *M* NaI solution. The conditions were identical for carboplatin but used 1.4 mg carboplatin.

### 2.2. X-ray diffraction data collection, structure solution and model refinement   

A crystal of HEWL with cisplatin or carboplatin was scooped into a loop with silicone oil used as the cryoprotectant. All X-ray diffraction (XRD) data were measured on a Bruker APEX II home-source diffractometer at an X-ray wavelength of 1.5418 Å at a fixed temperature of 100 K (Table 1 ▶), using an XRD data-collection strategy to obtain generally good data sets, *i.e.* high completeness of unique data, high anomalous difference completeness and good data redundancy. XRD data from both crystals were processed using the *SAINT* software package (v.2; Bruker AXS Inc., Madison, WI, USA).

The crystal structures were solved using molecular replacement with *Phaser* (McCoy *et al.*, 2007 ▶) followed by rigid-body refinement with *REFMAC*5 from *CCP*4 (Murshudov *et al.*, 2011 ▶) using the reported monoclinic lysozyme structure with PDB code 1lkr as the molecular search model (Steinrauf, 1998 ▶). Amplitude twin refinement was carried out for the cisplatin data set. Anomalous difference maps were generated using calculated phases with the ligands omitted from the model. These maps allowed a check for and identification of the I-atom positions. Model building, adjustment and restrained refinement were carried out using *Coot* (Emsley & Cowtan, 2004 ▶) and *REFMAC*5 (Murshudov *et al.*, 2011 ▶) in *CCP*4. Ligand-binding occupancies were calculated using *SHELXTL* (Sheldrick, 2008 ▶). The platinum-to-ligand distances were not restrained during refinement. The crystallographic and molecular model-refinement parameters are summarized in Table 1 ▶. Figures were prepared with *CCP4mg* (McNicholas *et al.*, 2011 ▶)

## 3. Results   

### 3.1. Cisplatin study   

**Figure 1:**
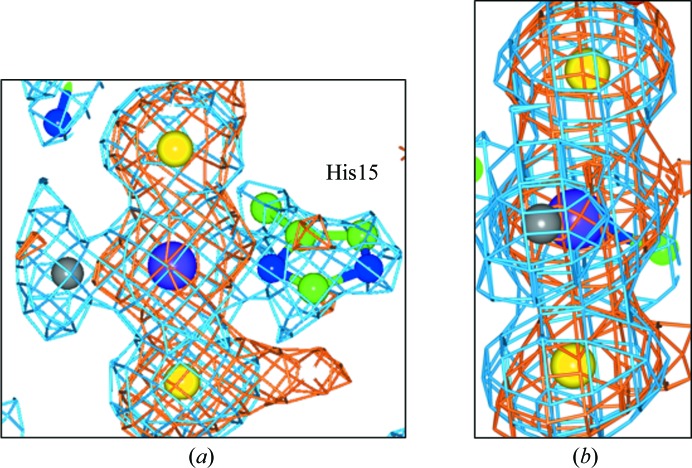
Binding to His15 shows a chemically transformed cisplatin, namely transiodoplatin. (*a*, *b*) The molecule *A* binding site shown in two different views. The 2*F*_o_ − *F*_c_ electron-density map (blue) is contoured at 1.5 r.m.s. and the anomalous difference electron-density map (orange) is contoured at 3σ. The Pt atom is shown in purple, the iodines are in yellow, the chlorine is in grey, C atoms are in green and N atoms are in blue.

Ligand binding is seen at His15 residue of both molecules *A* and *B* of HEWL in this monoclinic crystal form, *i.e.* with two molecules per asymmetric unit. In molecule *A* platinum binding is only seen at the N^δ^ binding site (Fig. 1 ▶), with an anomalous difference electron-density peak height of 9.8σ and an occupancy value of 80% (Table 2 ▶). Besides the platinum peak, there are two large anomalous difference electron-density peaks of 11.1σ and 8.8σ in the *trans* positions which are readily assignable as I atoms at distances of 2.6 Å (±0.1 Å) from the Pt atom, confirming that cisplatin has converted to the *trans* iodo-platinated form (transiodoplatin). The angle between these three atoms is 176°, which is close to linearity. A third 2*F*_o_ − *F*_c_ electron-density peak is seen bound to the Pt atom. However, no anomalous difference electron density is observed here and so a third I atom is ruled out. Modelling in an N atom from cisplatin gave a *B* factor of 2.0 Å^2^, which is physically unrealistic, and therefore a Cl atom was modelled in. This Cl atom has an occupancy of 100% as calculated by *SHELX* and a quite reasonable *B* factor of 21.3 Å^2^. The distance between the platinum and this chlorine is 2.4 Å (±0.1 Å) and can be compared with the usual platinum–chlorine distance of 2.35 Å; as this distance is well within the error of the bond-distance estimate (0.1 Å), this atom assignment seems reasonable. In molecule *B*, the identification of the compound bound to the N^δ^ atom of His15 (Supplementary Fig. S1^1^) is more difficult than in molecule *A* described above (Fig. 1 ▶) and the figure is given in the Supporting Information for completeness. Platinum binding is again only seen at the N^δ^ binding site (Fig. 1 ▶), with an anomalous difference electron-density peak height of 6.7σ and an occupancy of 67% (Table 2 ▶). There are also two other anomalous difference electron-density peaks in the binding site (7.2 and 4σ) in the *trans* position to the Pt atom and these are therefore assigned as I atoms bound to the platinum, each at a distance of 2.6 Å (±0.2 Å). The angle between these three atoms is 169°, showing signs of some distortion from linearity. In molecule *B* there are also two extra 2*F*_o_ − *F*_c_ density peaks close to the Pt and I atoms. Modelling in an N atom from cisplatin again gave a *B* factor of 2.0 Å^2^. Owing to this, a mixture of cisplatin and transiodoplatin appears to be bound to this N^δ^ atom. The distance between platinum and chlorine 1 is 2.4 Å (±0.3 Å) and the platinum–chlorine 2 distance is 2.8 Å (±0.1 Å). The occupancies of each molecule as a whole, estimated using *SHELX*, gave values of 51% for the transiodoplatin molecule and 49% for the cisplatin molecule. A third 2*F*_o_ − *F*_c_ electron-density peak is also seen, with no anomalous difference electron density observed at this position. An Na ion with 100% occupancy has been modelled in with a *B* factor of 33 Å^2^.

At the His15 N^∊^ binding sites in molecules *A* and *B*, an anomalous difference electron-density peak of 4.1σ and 4.0σ is seen, respectively, at 3.9 (±0.2) and 5.0 Å (±0.2 Å) from the N^∊^ atom, too distant to be a Pt atom. This peak is instead assigned as an I atom in both cases, which is 3.9 Å (±0.2 Å) from the NH backbone group of Ile88, which is in the correct distance range for a halogen hydrogen bond. This is the same situation as previously seen in the NaBr crystallization conditions case (Tanley, Diederichs *et al.*, 2014 ▶), where a Br atom was assigned at this position.

### 3.2. Carboplatin study   

Ligand binding is seen at the His15 N^δ^ atoms in both molecule *A* and molecule *B*. In molecule *A*, a platinum peak with an anomalous difference electron density of 9.8σ is seen which refines to an occupancy of 77% (Supplementary Fig. S2 and Table 2 ▶). Two further anomalous difference electron-density peaks are seen (10.8σ and 7.8σ) in the *trans* position to the Pt atom and these are assigned as I atoms bound to the Pt atom with a distance of 2.6 Å (±0.3 Å) (Supplementary Fig. S2). A third 2*F*_o_ − *F*_c_ electron-density peak is seen at a binding distance from the Pt atom, but no anomalous difference electron density is observed here. Owing to the lack of anomalous difference density, a portion of the CBDC moiety was modelled in. Thus, similar to the carboplatin in NaBr conditions (Tanley, Diederichs *et al.*, 2014 ▶), a portion of the CBDC moiety must still be present at this binding position.

**Figure 2:**
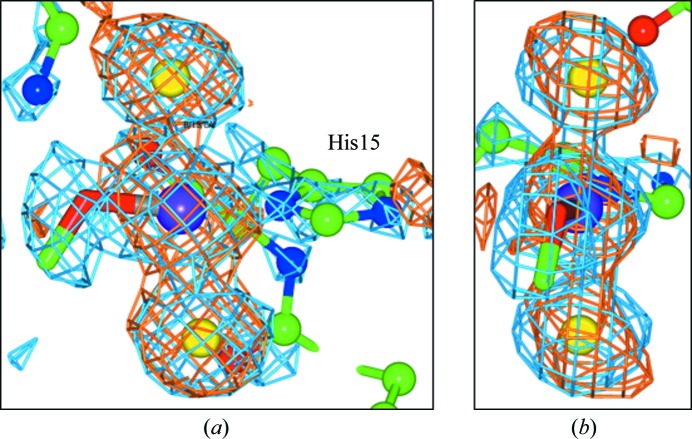
Binding of carboplatin chemically transformed to transiodoplatin to His15. (*a*, *b*) The molecule *B* binding site shown in two different views. The 2*F*_o_ − *F*_c_ electron-density map (blue) is contoured at 1.5 r.m.s. and the anomalous difference electron-density map (orange) is contoured at 3σ. The Pt atom is shown in purple, the iodines are in yellow, C atoms are in green, O atoms are in red and N atoms are in blue.

In molecule *B*, a platinum anomalous difference electron-density peak of 12.2σ is observed which refines to an occupancy of 68% (Fig. 2 ▶ and Table 2 ▶). Two anomalous difference density peaks are seen (12.1σ and 11.3σ) in *trans* positions to the Pt atom and these are assigned as I atoms bound to the Pt atom at a distance of 2.6 Å (±0.2 Å). A third 2*F*_o_ − *F*_c_ electron-density peak is seen at a binding distance to the Pt atom, with a weak anomalous difference electron-density peak of 2.6σ. Owing to the weakness of the peak it was not assigned to an atom type, but the 2*F*_o_ − *F*_c_ density is more elongated than in the *trans* iodo positions at this site and could correspond to a mixture of an I atom and a portion of the CBDC moiety (Fig. 2 ▶). Modelling in a mixture of an I atom and a portion of the CBDC moiety gives occupancies of 52% for the I atom and 48% for the CBDC moiety. Thus, the carboplatin has partially converted to the *trans* iodo-platinated form, with some evidence that a portion of the carboplatin molecule is still present. Molecule *B* is shown in Fig. 2 ▶ and molecule *A* in Supplementary Fig. S2 as they are very similar and the electron density around the carboplatin is slightly better for molecule *B*.

At the N^∊^ binding site in molecules *A* and *B* anomalous difference electron-density peaks of 3.4σ and 5.5σ are seen 5.1 (±0.4) and 5.5 Å (±0.2 Å) from the N^∊^ atom, too distant and too weak to be assignable as a Pt atom. This peak is assigned as an I atom in both cases which is 3.5 Å (±0.4 Å) and 3.9 Å (±0.2 Å) from the NH backbone group of Ile88, which is in the correct distance range for a halogen hydrogen bond.

### 3.3. A third Pt ligand-binding site in a crevice between two symmetry-related molecules   

**Figure 3:**
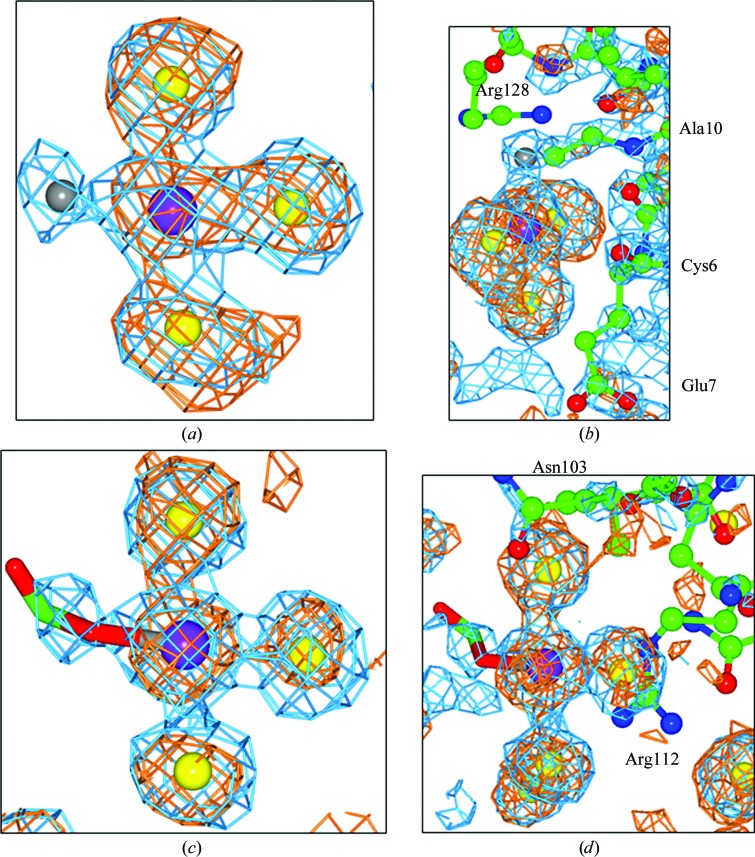
A PtI_3_ *X* complex bound in a crevice between two symmetry-related molecules. (*a*, *b*) Complex derived from cisplatin with a Pt atom bound to three I atoms and a Cl atom bound near Arg128, Cys6, Glu7 and Ala10 of molecule *A* and Asn106, Arg112, Pro70 and Asn65 of a symmetry-related molecule. (*c*, *d*) Complex derived from carboplatin with platinum bound to three I atoms and a portion of the CBDC moiety bound near Asn106, Arg112, Pro70, Asn65 of molecule *A* and Arg128, Cys6, Glu7 and Ala10 of a symmetry-related molecule (*a*). The 2*F*_o_ − *F*_c_ electron-density map (blue) is contoured at 1.5σ and the anomalous difference electron-density map (orange) is contoured at 3σ. The Pt atom is shown in purple, the iodines are in yellow, the chlorine is grey, C atoms are green and O atoms are red.

For both the cisplatin and carboplatin HEWL NaI crystal studies, a third platinum binding site is seen where a PtI_3_ moiety is identified bound in a crevice between two symmetry-related molecules near Asn106, Arg112, Pro70, Asn65 and Asn103 of molecule *A*, and Arg128, Cys6, Glu7 and Ala10 of a symmetry-related molecule. However, this binding is not seen at the same residues in molecule *B*. In the cisplatin case, three anomalous difference electron-density peaks of 9.4σ, 8.2σ and 7.8σ are seen (Table 2 ▶) at distances of 2.7 (±0.1), 2.5 (±0.1) and 2.5 Å (±0.1 Å) from the Pt atom, respectively. A fourth 2*F*_o_ − *F*_c_ electron-density peak is found at a distance of 2.4 Å (± 0.2 Å) from the Pt atom (Fig. 3 ▶ *a*). A Cl atom is assigned at this fourth position. In the carboplatin study, three anomalous difference electron-density peaks of 5.4σ, 5.9σ and 9.2σ are seen (Table 2 ▶) at distances of 2.7 (±0.3), 2.5 (±0.3) and 2.7 Å (±0.3 Å) from the Pt atom, respectively. A fourth 2*F*_o_ − *F*_c_ electron-density peak within binding distance of the Pt atom has elongated density (Fig. 3 ▶ *b*). We interpret this as a portion of the CBDC moiety of carboplatin bound at this fourth position.

## 4. Discussion   

Our studies have investigated whether co-crystallizing cisplatin or carboplatin with HEWL in NaI conditions, rather than using NaCl, would result in partial or full chemical conversion of these compounds to the iodo form. Previous results with carboplatin in NaCl crystallization conditions showed partial conversion to cisplatin (Tanley *et al.*, 2013 ▶), whereas in sodium bromide conditions the carboplatin was partially converted to the *trans* bromo form with a portion of the CBDC moiety still being present (Tanley, Diederichs *et al.*, 2014 ▶). Obviously, cisplatin with HEWL under sodium chloride conditions would simply preserve chlorinated platinum in the cisplatin.

The results presented here for both cisplatin and carboplatin indeed showed partial conversion to the *trans* iodo form with a portion of a Cl or CBDC moiety still seen in the binding site (Figs. 1 ▶ and 2 ▶), which is similar to the results using NaBr as the crystallization high-salt component (Tanley, Diederichs *et al.*, 2014 ▶). Tanley, Diederichs *et al.* (2014 ▶) describe carboplatin binding to HEWL in NaBr crystallization conditions as well as in non-NaCl and non-NaBr conditions, the latter being in order to see a chemically unmodified carboplatin. A study of cisplatin binding to HEWL in NaBr conditions has also been carried out, and for completeness those results are given in the Supporting Information to this paper. The results are very similar to the carboplatin study in NaBr conditions, in which the cisplatin is also seen to be converted to a transbromoplatin form with a Cl atom also bound to the platinum centre at the N^δ^ binding site. Similar to the carboplatin study, the N^∊^ binding site shows less detail, with a weakly occupied Pt atom (20%) bound to a Br atom.

Previously, under NaCl conditions at pH 4.7, we have seen clear binding at both the N^δ^ and N^∊^ sites of His15. This was attributed to the chlorine extracting a His15 H atom, creating an imidazolyl ion (Tanley *et al.*, 2012 ▶). In the bromo case (Tanley, Diederichs *et al.*, 2014 ▶) binding at both sides was still seen, but with a much weaker occupancy (up to 20%) for the N^∊^ binding site. We rationalize this as being owing to bromine ions being weaker than chloride ions at extracting the last His H atom to generate the imidazolyl ion. Another possibility is tautomeric forms; see Tanley *et al.* (2012 ▶) for a full discussion. Where binding is seen at only one histidine N atom we are obviously not seeing a tautomeric pair or an imidazolyl anion. In the iodo case, the hydrogen-abstracting effectiveness is worse than for bromide ions and this is a likely reason why the binding of a platinum ligand is seen only at one His15 N atom, at least at pH 4.7, in the NaI case. A further difference between the NaI conditions and the NaCl or NaBr conditions was the fact that the crystals are monoclinic, rather than tetragonal, and thereby had two protein molecules in the asymmetric unit. In both monoclinic asymmetric units we note that binding only occurs to the N^δ^ atom of His15.

Recently, Messori *et al.* (2013 ▶) soaked PtI_2_(NH_3_)_2_ into pre-grown HEWL crystals and the Pt atom was seen to be bound to the N^δ^ atom of His15 but, as they described it, showed ‘peculiar features’ involving

the presence of three peaks of anomalous electron density close to a Pt atom suggesting the presence of two alternative modes of binding of the [PtI_2_NH_3_] moiety.

In our studies reported here, by using NaI in the crystallization mixture we examined whether the cisplatin or carboplatin was converted to an iodo form with I atoms bound to the platinum centre. For our NaI co-crystallization conditions we see a PtI_2_ *X*_2_ species at His15, *i.e.* transiodoplatin bound to the His15 N atom. In addition, though, we also see a third platinum moiety, a PtI_3_ *X* species, which is bound in a crevice between molecules *A* through residues Asn106, Arg112, Pro70, Asn65 and Asn103, and Arg128, Cys6, Glu7 and Ala10 of a symmetry-related molecule. This binding is not seen at the same residues for molecule *B*. The evidence that a platinum is seen bound to three I atoms is based on the presence of anomalous difference electron-density peaks (Fig. 3 ▶) and then on their refined occupancies. The fourth bound atom, *i.e.* the *X* in PtI_3_ *X*, is interpreted as a Cl atom (cisplatin) or a portion of the CBDC moiety (carboplatin). This is reminiscent of Zeise’s salt PtCl_3_C_2_H_4_ (Black *et al.*, 1969 ▶).

Finally, we note that in our companion paper on HEWL with Pt hexahalides we see a PtI_3_ species bound to the His15 N atom (Tanley, Starkey *et al.*, 2014 ▶).

## 5. Conclusions   

Co-crystallization of HEWL with cisplatin and carboplatin in NaI was carried out, with the XRD results showing that both platinum compounds indeed underwent a partial chemical conversion. In both cases this was to transiodoplatin (*i.e.* PtI_2_ *X*_2_) bound to the His15 N atom, with either a Cl atom (cisplatin) or a portion of the CBDC moiety (carboplatin) bound at the fourth Pt ligand atom position. These results are similar to our previous results using NaBr crystallization conditions. In the NaI conditions, only binding to the N^δ^ atom of His15 is seen; this is attributed to the inability of iodide ions to produce an imidazolyl ion, at least at the pH used in these experiments.

A PtI_3_ *X* species is seen bound in a crevice between symmetry-related protein molecules; here the platinum is bound to three I atoms based on the presence of anomalous difference electron density and their refined occupancies, with the fourth bound atom being a Cl atom (cisplatin) or a portion of the CBDC moiety (carboplatin). The observation of these iodinated platinum species suggests the prospect of undertaking a dual *K*-edge (platinum and iodine) targeted radiation therapy experiment against a tumour. Cisplatin administration has previously been studied in this way involving radiation therapy using a synchrotron source and the Pt *K* edge alone (Biston *et al.*, 2004 ▶; see also http://www.esrf.eu/UsersAndScience/Publications/Highlights/2004/Imaging/Ima10/). To undertake such a dual *K*-edge radiation therapy involving iodinated cisplatin or carboplatin under patient conditions would build upon experiments such as are already under way at the ESRF with cisplatin as cited above.

## 6. Related literature   

The following reference is cited in the Supporting Information for this article: Cianci *et al.* (2008 ▶).

## Supplementary Material

PDB reference: carboplatin/NaI, 4owa

PDB reference: cisplatin/NaBr, 4owb

PDB reference: cisplatin/NaI, 4ow9

Supporting Information.. DOI: 10.1107/S2053230X14013995/no5053sup1.pdf

## Figures and Tables

**Table 1:** X-ray crystallographic data and final protein plus ligand model-refinement statistics for both the cisplatin and carboplatin crystals Values in parentheses are for the last shell.

	Cisplatin/NaI	Carboplatin/NaI
PDB code	4ow9	4owa
Data-collection temperature (K)	100	100
Data reduction		
Space group	*P*2_1_	*P*2_1_
Unit-cell parameters (Å, °)	*a* = 27.25, *b* = 62.54, *c* = 58.98, β = 90.92	*a* = 27.14, *b* = 62.28, *c* = 58.05, β = 92.59
Molecules per asymmetric unit	2	2
Observed reflections	110261	104255
Unique reflections	11636	20257
Resolution (Å)	31.27–2.10 (2.15–2.10)	29.04–1.80 (1.90–1.80)
Completeness (%)	90.1 (55.7)	94.3 (78.0)
*R*_merge_ (%)	0.130 (0.229)	0.099 (0.340)
〈*I*/σ(*I*)〉	11.7 (3.5)	11.7 (1.9)
Multiplicity	6.9 (1.6)	5.7 (1.4)
Refinement		
Cruickshank DPI (Å)	0.11	0.18
No. of atoms		
Protein	2002	2002
Water molecules	39	147
Pt and halogen atoms	34	42
Other bound atoms	28	28
Average *B* factor (Å^2^)		
Protein atoms	21.1	12.2
Water molecules	18.8	19.2
Pt and halogen atoms	29.1	20.9
Other bound atoms	24.8	18.4
*R* factor/*R*_free_ (%)	19.7/27.1†	18.4/24.0
R.m.s.d., bonds (Å)/angles (°)	0.010/1.5	0.015/1.6
Ramachandran values (%)		
Most favoured	94.1	96.5
Additional allowed	5.2	3.1
Disallowed	0.8‡	0.4‡

† The PDB validation report includes *phenix.xtriage* statistics; subsequent use of *DETWIN* in *CCP*4 suggested that the data were 6% twinned. To our knowledge, no twinning has been reported for this form of HEWL in NaI conditions before. Turning on amplitude twin refinement in *REFMAC* improved the *R*/*R*_free_ from 19.7/27.5% to 19.7/27.1%. The electron-density maps at the His binding sites as well as in general were the same with and without using twin refinement, presumably owing to the rather small twin fraction.

‡ The disallowed residues are Asp18, Ser72 and Arg73 and are part of a loop/turn region.

**Table 2:** Occupancy values of the Pt and I atoms for both the cisplatin and carboplatin cases calculated using *SHELX*

		Cisplatin study		Carboplatin study	
		Anomalous peak height (σ)	Occupancy (%)	Anomalous peak height (σ)	Occupancy (%)
Pt	His15 N^δ^ binding site, molecule *A*	9.8	80 ± 12	9.8	77 ± 5
I1		11.1	86	10.8	70
I2		8.8	89	7.8	86
Pt	His15 N^δ^ binding site, molecule *B*	6.7	67 ± 11	12.2	68 ± 4
I1		7.2	77	12.1	70
I2		4.0	67	11.3	77
Pt	In a crevice between two symmetry-related molecules near Asn106	6.7	57	8.8	68
I1		9.4	60	5.4	70
I2		8.2	74	5.9	77
I3		7.8	62	9.2	69

## Footnotes

1 Supporting information has been deposited in the IUCr electronic archive (Reference: NO5053).
