# Supplementary material for: Chemical conversion of cisplatin and carboplatin with histidine in a model protein crystallized under sodium iodide conditions
Source: Acta Crystallogr F Struct Biol Commun. 2014 Aug 29;70(Pt 9):1127–31. doi: 10.1107/S2053230X14013995 (PMC4157406; doi:10.1107/S2053230X14013995)
Supplement: Supplementary file 1 [file f-70-01127-sup1.pdf]

Acta Crystallographica Section F

Volume 70 (2014)

Supporting information for article:

Chemical conversion of cisplatin and carboplatin with histidine in a model protein crystallised under sodium iodide conditions

Simon W. M. Tanley and John R. Helliwell

S1. Cisplatin binding to His-15

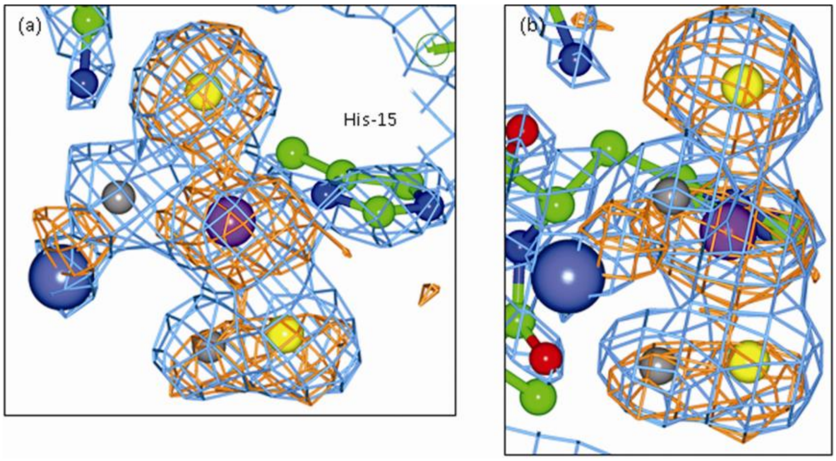

Figure S1 (a) and (b) His-15 binding site in molecule B shown in two different views showing the split occupancy transiodoplatin and cisplatin molecules (this interpretation is more complicated and for details see text). The 2Fo-Fc electron density map (blue) is contoured at 1.5σ and the anomalous difference electron density map (orange) is contoured at 3σ . The platinum atom is shown in purple, iodine atoms in yellow, chlorine atoms in grey and sodium atom in light blue, carbons are in green, nitrogens are in blue and oxygens are in red.

S2. Carboplatin binding to His-15

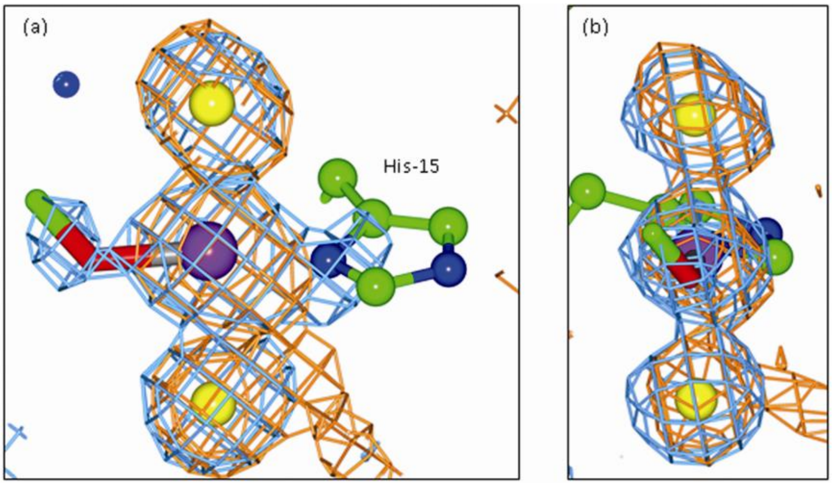

Figure S2 (a) and (b) His-15 binding site in molecule A shown in two different views. The 2Fo-Fc electron density map (blue) is contoured at 1.5σ and the anomalous difference electron density map (orange) is contoured at 3σ . The platinum atom is shown in purple, the iodines are in yellow, the chlorines are in grey, the carbons are in green, nitrogens are in blue and oxygens are in red.

S3. Cisplatin binding to HEWL in NaBr crystallisation conditions

20mg HEWL was co-crystallised with 1.2mg cisplatin with 75µl DMSO, 462.5µl 0.1M NaAc and 462.5µl 1M NaBr solution. A crystal was scooped into a loop with silicon oil used as the cryoprotectant, and XRD data measured on a Bruker APEXII home source diffractometer at an X-ray wavelength of 1.5418Å, carried out at a fixed temperature of 100K (Table S1) with an XRD data collection strategy used to gain generally good datasets i.e. high completeness of unique data, high anomalous differences completeness and good data redundancy. The XRD data were processed using the Bruker software package (SAINT).

The crystal structures were solved using molecular replacement with PHASER (McCoy *et al*, 2007) and then rigid body refinement with CCP4i REFMAC5 (Vagin *et al*, 2004), using the reported lysozyme structure 2W1Y as the molecular search model (Cianci *et al*, 2008). Anomalous difference density maps were calculated with calculated phases with the ligands omitted from the model. These maps allowed a check for and identification of the iodine atom positions. Model building, adjustment and restrained refinement were carried out respectively using the COOT (Emsley & Cowtan, 2004) molecular graphics programme and REFMAC5 (Vagin, 2004) in CCP4i. Ligand binding occupancies were calculated using SHELXTL (Sheldrick, 2008). The crystallographic and molecular model refinement parameters are summarized in Table S1.

Table S1 X-ray crystallographic data and final protein plus ligand model refinement statistics.

	Cisplatin NaBr
PDB id	4OWB
Data collection temperature (K)	100
**Data reduction**	
Space group	$P4_32_12$
Unit cell parameters (Å)/(°)	a=b= 78.15<br>c= 37.43
Detector to crystal distance (mm)	40.00
Observed reflections	739708
Unique reflections	13073
Resolution (Å) (last shell)	30.99- 1.69 (1.79-1.69)

Completeness (%)	99.8 (98.7)
Rmerge (%)	0.155 (0.661)
(I/σ(I))	29.7 (1.5)$
Multiplicity	55.1 (9.5)
Cruickshank DPI (Å)	0.132
Number of protein atoms	1001
Average B factor ($Å^2$) for protein atoms	19.7
Number of water molecules	103
Average B factor ($Å^2$) for water molecules	26.7
Number of Pt and Br atoms	11
Average B factor ($Å^2$) for Pt and I atoms	32.9
Other bound atoms	21
Average B factor ($Å^2$) for other bound atoms	27.4
**Refinement**	
R factor/ R free	20.4/26.6
R factor all	20.7
RMSD bonds (Å)/ Angles (°)	0.01/1.4
**Ramachandran values (%)**	
Most favoured	95.3
Additional allowed	4.7
Disallowed	0

$ (I/σ(I))crosses 2 at 1.79Å.

In the Nδ binding site (Figure S3), a platinum centre is seen bound to two bromine atoms in the trans position. Anomalous difference electron density is only seen at one of these positions, but the 2Fo-Fc electron density peaks are of similar shape and height to that of the carboplatin crystallised in NaBr conditions. Thus, the partial conversion to the trans bromo form in the cisplatin case is also thought to take place. The third binding site to the platinum centre is harder to interpret, but based on the 2Fo-Fc density peak, a chlorine atom has been modelled in, in a similar way to the conversion of cisplatin to the transiodoplatin form in NaI crystallisation conditions (Tanley & Helliwell, 2014), where a chlorine atom is bound at the third positions. The Nε binding site is harder to interpret, but in the same

way as the carboplatin dataset (Tanley *et al*, 2014), due to the distances from the Nε atom, a platinum atom is placed bound to the Nε atom and to a bromine atom.

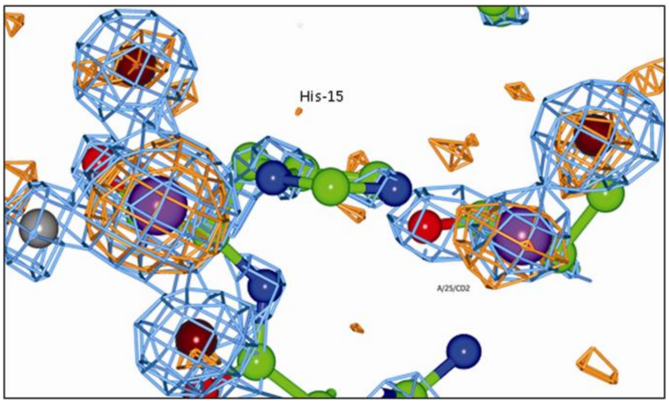

Figure S3 Cisplatin binding to His-15 of HEWL. The 2Fo-Fc electron density map (blue) is contoured at 1.5σ and the anomalous difference electron density map (orange) is contoured at 3σ. Platinum atoms are in purple, bromine atoms in dark red and chlorine atom in grey.

Cianci, M., Helliwell, J. R. & Suzuki, A. (2008). *Acta Cryst*. *D*64:1196–1209.

Emsley, P. & Cowtan, K. (2004). *Acta Cryst*. *D***60**, 2126–2132.

McCoy, A. J., Grosse-Kunstleve, R. W., Adams, P. D., Winn, M. D., Storoni, L. C. & Read, R. J. (2007). *J. Appl. Cryst*. **40**, 658–674.

Sheldrick, G.M. (2008). *Acta Cryst*. A**64**, 112-122

Tanley S.W.M, Schreurs AMM, Kroon-Batenburg LMJ, Meredith J, Prendergast R, Walsh D, Bryant P, Levy C, Helliwell JR (2012). *Acta Cryst D* **68**:601-612

Tanley, S.W.M, Diederichs, K, Kroon-Batenburg, L.M.J, Levy, C, Schreurs, A.M.M and Helliwell, J.R (2014) Submitted to Acta F

Vagin, A. & Teplyakov, A. (2010). *Acta Cryst.* D**66**, 22–25.
